# “Medically unexplained” symptoms and symptom disorders in primary care: prognosis-based recognition and classification

**DOI:** 10.1186/s12875-017-0592-6

**Published:** 2017-02-07

**Authors:** Marianne Rosendal, Tim C Olde Hartman, Aase Aamland, Henriette van der Horst, Peter Lucassen, Anna Budtz-Lilly, Christopher Burton

**Affiliations:** 1 Research Unit for General Practice, Department of Public Health, Aarhus University, Bartholins Alle 2, DK-8000 Aarhus C, Denmark; 2 Research Unit of General Practice, Institute of Public Health, University of Southern Denmark, J.B. Winslows Vej 9 A, DK-5000 Odense, Denmark; 3 Department of Primary and Community Care, Radboud University Nijmegen Medical Center, Nijmegen, The Netherlands; 4 Research Unit for General Practice, Uni Research Health, Bergen, Norway; 5 Department of General Practice and Elderly Care Medicine, VU University Medical Centre, Amsterdam, The Netherlands; 6 Academic Unit of Primary Medical Care, University of Sheffield, Samuel Fox House, Northern General Hospital, Sheffield, S5 7 AU UK

**Keywords:** Somatoform disorders, Signs and symptoms, Symptom assessment, General practice, Primary health care, Classification (non-MESH), Diagnosis (non-MESH), Symptom research (non-MESH), Medically unexplained symptoms (non-MESH)

## Abstract

**Background:**

Many patients consult their GP because they experience bodily symptoms. In a substantial proportion of cases, the clinical picture does not meet the existing diagnostic criteria for diseases or disorders. This may be because symptoms are recent and evolving or because symptoms are persistent but, either by their character or the negative results of clinical investigation cannot be attributed to disease: so-called “medically unexplained symptoms” (MUS).

MUS are inconsistently recognised, diagnosed and managed in primary care. The specialist classification systems for MUS pose several problems in a primary care setting. The systems generally require great certainty about presence or absence of physical disease, they tend to be mind-body dualistic, and they view symptoms from a narrow specialty determined perspective. We need a new classification of MUS in primary care; a classification that better supports clinical decision-making, creates clearer communication and provides scientific underpinning of research to ensure effective interventions.

**Discussion:**

We propose a classification of symptoms that places greater emphasis on prognostic factors. Prognosis-based classification aims to categorise the patient’s risk of ongoing symptoms, complications, increased healthcare use or disability because of the symptoms. Current evidence suggests several factors which may be used: symptom characteristics such as: number, multi-system pattern, frequency, severity. Other factors are: concurrent mental disorders, psychological features and demographic data. We discuss how these characteristics may be used to classify symptoms into three groups: self-limiting symptoms, recurrent and persistent symptoms, and symptom disorders. The middle group is especially relevant in primary care; as these patients generally have reduced quality of life but often go unrecognised and are at risk of iatrogenic harm. The presented characteristics do not contain immediately obvious cut-points, and the assessment of prognosis depends on a combination of several factors.

**Conclusion:**

Three criteria (multiple symptoms, multiple systems, multiple times) may support the classification into good, intermediate and poor prognosis when dealing with symptoms in primary care. The proposed new classification specifically targets the patient population in primary care and may provide a rational framework for decision-making in clinical practice and for epidemiologic and clinical research of symptoms.

## Background

Many patients consult their general practitioner (GP) because they experience bodily symptoms. Western medicine prioritises the assessment of symptoms to diagnose disease, but symptoms are not exclusively signs of disease [1]. Some symptoms represent ordinary bodily sensations causing minor concern [2, 3], others arise, or persist, due to processes which do not depend on underlying disease [4]. When symptoms persist but, either by their character or the negative results of clinical investigation, cannot be attributed to disease, they are commonly described as “medically unexplained symptoms” (MUS).

Understanding, recognising, explaining and managing MUS are core tasks in general practice. Nevertheless, many practitioners are challenged by these tasks [5], not least because conceptualisations of the problem are unclear and vary between doctors [6–8]. Professional assessment, communication and treatment are based on knowledge of disease patterns and such patterns may be described by classification criteria. This knowledge is also a precondition for communication with patients in order to provide a reliable basis for their subsequent actions. Furthermore, research in this field is hampered by inconsistent criteria and would profit considerably if we can reach consensus on a classification which is useful in primary care [8].

This debate paper aims to describe why classification of MUS in primary care is difficult - but important - and proposes a shift in focus towards prognostic classification.

### What causes MUS, if not disease?

Bodily symptoms arise from both peripheral and central processes [4]. While disease-based medicine has focused on peripheral triggers (i.e., disease or injury in an organ), recent work has demonstrated the importance of central symptom processing [9, 10]. “Central sensitisation” is an example of central processes involved in the persistence or amplification of symptoms. This was first elaborated in relation to pain, but also appears to play a role for other symptoms [9].

Symptom processing can be considered at the psychological level (as described in cognitive-behavioural models of MUS [11]) and increasingly also at the neuro-physiological level [12]. Furthermore, altered central symptom processing may give rise to increased peripheral symptom production (e.g., autonomic arousal). Previous models of MUS have emphasized the idea of “somatisation”, i.e., the presence of bodily symptoms as indirect markers of psychological distress [13]. Although considerable comorbidity has been found between both moderate and severe MUS and common mental disorders, the idea of direct psychological causality to symptoms is regarded too simplistic to account for most MUS [14].

In this paper we will use the term MUS to refer to symptoms which are primarily influenced by central processes rather than peripheral organ disease or injury. Some clinicians and researchers use the term “functional symptoms” instead of MUS, but this is still used disparagingly by some doctors and is probably not yet suitable for widespread use in primary care settings. Additionally, most medical specialties have clusters of MUS within so-called ‘functional syndromes’ (e.g., Fibromyalgia, Irritable Bowel Syndrome) [15]. We recognise that there is no single ideal term here and patients prefer either specific syndrome labels (such as Fibromyalgia) or generic terms including the word ‘physical’ as e.g., persistent physical symptoms [16, 17]; however most primary care physicians and researchers are familiar with the umbrella term MUS and so we will continue to use it for now.

### MUS in primary care

One in three consultations in primary care is concluded without specific diagnosis [18] and approximately one in six primary care consultations involve MUS [19, 20]. Yet, most patients consulting with MUS do so only sporadically. Around 3–10% of adult GP consulters have persistent or recurring MUS [21–24]. This is associated with reduced health-related quality of life, increased healthcare use and increased prevalence of depression and anxiety [24–26]. Furthermore, patients with MUS have increased risk of drop-out from the labour market [27].

There is good evidence that patients with MUS do not get sustained reassurance from negative diagnostic tests or medical opinions [28]. Despite this, both patients and doctors may be trapped in a situation where they seek to use a biomedical disease model in their search for cause and explanations [29] because the alternatives involve conflicts of real and perceived beliefs about the nature of MUS. Consequently, MUS challenge both the GP and the doctor-patient relationship [5, 30]; and many doctors have negative attitudes towards patients with severe MUS [31, 32]. Furthermore, patients with persistent symptoms are at risk of iatrogenic harm as they may go through numerous investigations and receive unnecessary treatment such as medication and surgery [33–35]. Finally, patients with MUS are generally less satisfied with their encounters than patients with biomedical diseases [36, 37] and may even feel rejected by their GP [38, 39].

### Why do we need to classify MUS in primary care?

Classification of MUS in primary care is needed for three reasons: for explanations to patients, for clinical decision-making and for research.

For patients, the desire to make sense of symptoms has been demonstrated repeatedly in studies of patients with MUS [40–43]. Classification can act as a starting point for an explanation which may lead to treatment or support for self-management [44].

For professionals and health services, classifications assist the clinical decisions on management, in particular whether or not to pursue (further) clinical investigation. Additionally, classification provides a shared language for communication between professionals.

In research, consensus on classification provides consistent entry criteria for epidemiological studies and clinical trials; these are necessary to explore illness course and to assess the effectiveness of interventions.

### Why is classifying MUS so difficult in primary care?

From our experience with research and teaching of GPs, we recognise three major problems relating to classification of MUS in primary care: high clinical uncertainty, mind-body dualism and the position of primary care between different perspectives on classification of MUS.

#### Primary care is a field of high clinical uncertainty

Primary care medicine is at the front line of the healthcare system and consequently faces a high degree of uncertainty when symptoms are first presented. This uncertainty may arise from several sources: many patients with MUS also develop (or already have) “explained” conditions, primary care clinicians often see patients before conditions or symptom patterns have fully developed, which makes it difficult to exclude organic disease, and the generalists in primary care are aware that they have less knowledge of uncommon medical conditions than medical specialists.

All of these sources of uncertainty imply that GPs tend to be cautious about firmly classifying patients as having MUS [7, 23]. Therefore, any classification system for MUS needs to be sufficiently flexible to accommodate the fact that some (not most) patients with suspected MUS will turn out to have underlying disease [45]. In other words, classifications and recommendations for general practice must allow GPs to “ride two horses” from the first meeting with the patient to ensure that both pathological and “medically unexplained” causes for symptoms are considered in parallel.

#### Most doctors learned classifications which locate the problem in the body or the mind, not both.

Medicine has a long tradition of mind-body dualism, which is no longer tenable in the light of current thinking and knowledge about the integrated nature of brain and body [46]. On the one hand, medical training and the legal system both put major emphasis on the importance of preventing delayed diagnosis of biomedical diseases, so emphasising the biological aspect. On the other hand, general practice has been heavily influenced by psychological perspectives through writers like Balint [47] who emphasise psycho-social causes of illness. Although approaches such as the bio-psycho-social model [1] seek to unite the mind and body in relation to illness, in clinical practice this remains problematic.

GPs are taught to look for reasons that go beyond the symptom: Why this? Why now? As we answer these questions, psychosocial factors come into view. Psychological factors can almost always be found if we look hard enough; they may be the true causes for symptoms, as predisposing and/or triggering factors, or they may be incidental. As diagnosticians, we learn to value the simplest formulation (whether it be biological or psychosocial). So, while patients with MUS recognise the role of multiple factors in their symptoms [48, 49], the GP’s instinct for simple formulations might imply that s/he misses the opportunity to integrate identified components in a way that is acceptable to the patients [50].

#### Primary care sits between different perspectives on diagnosis of MUS

The third reason why classification of MUS in primary care is difficult comes from the tension between two very different perspectives on classification: within individual medical specialties and from psychiatric epidemiology. The single medical specialty perspective pays most attention to specific symptom characteristics within one organ system. Thus, it pays less attention to symptoms in other bodily systems or to psychological characteristics. An example of a single speciality classification is the Rome Diagnostic Criteria for Functional Gastrointestinal Disorders (a common type of MUS); it specifies symptom characteristics and duration, but it does so only in relation to the gastro-intestinal tract [51]. On the other hand, psychiatric classifications pay less attention to the characteristics of specific symptoms, and most attention to the overall pattern of symptoms. Such classifications include number of symptoms (total or “unexplained”), presence of psychological criteria (e.g., excessive distress) or stipulated level of healthcare use. They continue to be challenged [52, 53].

GPs deal with both the narrow perspective of the current (reason for encounter) symptoms and the wider perspective of the whole patient. For example, a woman with abdominal pain and bloating may have both typical features of irritable bowel syndrome (IBS) and also additional symptoms such as tiredness, concentration difficulties and dizziness, which render her unable to work. The following vignette illustrates a typical clinical case:

*Anna is a 38-year-old office worker. She consults her GP for the third time in two months about abdominal pain and bloating, which are both worse after meals and have been present for several months. Similar symptoms occurred a year ago for around four months, but then disappeared for some months. She has no “red flags” for serious disease, and the routine blood tests (including antibodies for coeliac disease and lactose intolerance) are normal. She has stopped going out for meals with friends because of her symptoms. She also experiences dizziness (without vertigo), fatigue and difficulty concentrating. She has been unable to go to work for the last three weeks because of her symptoms. She describes her current symptoms as being “always in the back of her mind”, even on days when she is feeling better.*

*In the last three years, she has consulted her GP with palpitations (a 24-hour ECG showed sinus tachycardia) and pelvic pain. She has occasional migraine and more frequent milder tension-type headaches, which occur two or three days per week at work. Nevertheless, she “keeps going” and bringing up her young family with her husband, who works as an engineer in a factory which is currently under threat of closure.*

If seen by a gastroenterologist, Anna has IBS. If seen by a psychiatrist, she has somatic symptom disorder (SSD). If seen by a GP, she may have both and yet may be diagnosed with neither, and the GP may wonder if she needs more medical tests or a referral for psychological support. General practice thus sits in a “zone of generalism” between the medical specialty classification focusing on the nature of symptoms within a single organ system and the psychiatric classification focusing on numbers of symptoms rather than their nature. This zone of generalism is characterised by the uncertainty that is inherent in primary care medicine, but also by the uncertainty related to deciding which type of diagnostic classification is the most appropriate (or useful).

### Discussion: moving from diagnosis to prognosis as a basis for classification

We have so far argued that there is a need for classification of MUS. We have also argued that the current diagnostic classification is problematic in several ways: the inevitable residual uncertainty, the problems of cause-related mind-body dualism, and the zone of generalism positioned between medical specialties and psychiatry.

In the remainder of this article, we will propose a solution that draws on recent thinking about prognostic classification.

### A new prognosis-based classification

Classification of disease on the basis of prognosis does not seek to state definitively whether a patient does or does not have a condition. Rather, prognosis-based classification aims to categorise the patient’s risk of ongoing symptoms, complications or increased healthcare use because of the condition [54, 55]. An example comes from type 2 diabetes where a prognosis-based classification is less concerned with whether a patient meets the arbitrary criteria for diabetes than whether the prognosis or risk of future events is affected. Thus, two patients may both have a blood glucose level just above the threshold for diabetes. Yet, this may have different relevance if one is an obese 33-year-old (for whom it is a highly significant prognostic factor) and the other is an 88-year-old with dementia in a care home (for whom it is irrelevant).

Similarly, prognosis-based classification of MUS in primary care should not just consider whether a given symptom could be explained or unexplained, but rather should assess whether the symptom is likely to persist, recur or seriously impact the patient’s quality of life or future healthcare use.

Introducing prognosis-based classification of MUS in primary care does not exclude the use of functional syndrome labels (e.g., IBS, fibromyalgia), but rather serves to supplement these labels. We believe that positively categorising based on prognosis could help the GPs to better recognise when to concentrate on explaining and managing symptoms – as disorders of symptom processing – rather than continue the search for an organic diagnosis when all the pointers are in the opposite direction.

In terms of classification of MUS in primary care, we propose a classification with three prognostic categories. These three categories are based on current evidence about prognosis and the different needs for interventions depending on illness severity as described below. We call these categories: “Self-limiting symptoms”, “Recurrent or persistent symptoms” and “Symptom disorder”.

#### Self-limiting symptoms

Patients belonging in the self-limiting symptoms category have a good prognosis [56]. Their symptoms are relatively infrequent and unobtrusive. They occasionally seek healthcare for symptoms which appear not to be due to disease, and consultations for symptoms are usually single rather than repeated.

#### Symptom disorder

On the other hand, patients in the symptom disorder category have a poor prognosis. They have multiple symptoms with substantial symptom related disability and healthcare use. They commonly meet the clinical criteria for psychiatric classification disorders, such as SSD [57], or the research criteria for bodily distress syndrome (BDS) [58, 59]. Additionally, they may also have comorbid emotional disorders [26]. For these relatively few patients, we recommend that GPs consider using specific diagnoses in accordance with the criteria for psychiatric disorders and functional syndromes.

#### Recurrent or persistent symptoms

This leaves a prognostic category between these two extremes which we term “recurrent or persistent symptoms”. Patients in this middle category have repeated (although not necessarily frequent) symptoms for which they consult. These symptoms tend to persist for longer than patient or physician would expect, they are associated with reduced quality of life, and they may include a mix of unexplained and explained conditions. Patients with “recurrent or persistent symptoms” are much more common than patients with poor prognosis, but they often go unrecognised in primary care as they are mistaken for having (yet undetected) physical disease [45]. The reasons for this are not fully clear, but it may be because GPs lack a commonly used classification term for this group as they reserve MUS labels for patients in the more severe “symptom disorder” category. Consequently, we do not communicate about these patients as an independent group in need of specific management [7]. Our choice of label includes “persistent symptoms” because this term has been found more acceptable to patients than other labels for functional symptoms or syndromes [16].

### What information is needed for prognostic classification?

The prognosis for the patient – whether the symptoms will resolve, persist or increase (in time, scope, severity or impact) – can be influenced by a range of factors. We will here focus on the factors that can be readily elicited in a GP consultation or from primary care records and we present them grouped in themes.

### Symptom characteristics

#### Number of symptoms

Prospective studies have repeatedly demonstrated the value of “number of symptoms” as a predictor of poor health status in long-term follow-up studies [60–62]. This is true for symptoms in general, for MUS [63] and for somatoform disorders [64].

In the case of musculoskeletal pain, the number of pain-affected bodily sites is also a predictor of poor outcome in terms of disability [65, 66]. Finally, number of symptoms and number of pain sites have been shown to predict work disability [27, 67, 68].

#### Multi-system patterns of symptoms

Earlier studies widely used the total number of symptoms, whereas recent work has pointed to the value of including patterns of symptoms in multiple body systems. This is consistent with research demonstrating large overlaps between symptoms of different functional syndromes in severe conditions [69, 70]. Recent studies on BDS suggest that central sensitisation not only results in multiple symptoms; it may also prompt several specific symptom patterns described by arousal and/or exhaustion symptoms [58, 59, 70]. These symptoms cluster in four groups: 1) cardiopulmonary/autonomic arousal symptoms (palpitations/heart pounding, precordial discomfort, breathlessness without exertion, hyperventilation, hot or cold sweats, dry mouth), 2) gastrointestinal arousal symptoms (abdominal pains, frequent loose bowel movements, feeling bloated/full of gas/distended, regurgitations, diarrhoea, nausea, burning sensation in chest or epigastrium), 3) musculoskeletal tension symptoms (pains in arms or legs, muscular aches or pains, pains in the joints, feelings of paresis or localized weakness, backache, pain moving from one place to another, unpleasant numbness or tingling sensations), and 4) general symptoms (concentration difficulties, impairment of memory, excessive fatigue, headache, dizziness).

Patients with BDS have a high risk of poor quality of life, medicalisation and long-term persistence [25, 68, 71]. Interestingly, one study on MUS also found autonomic sensations to be an indicator of poor outcome [72].

Hence, symptom patterns of central sensitization as for example those seen in BDS may be a way forward to identify the most severely affected group of patients with symptom disorders [73].

#### Duration and frequency of symptoms

Duration and frequency of symptoms are included in certain specialist classifications (e.g., the Rome Diagnostic Criteria for Functional Gastrointestinal Disorders [51]), but the value of this is less clear in primary care and few intervention studies present data on the duration of symptoms at patient inclusion [63]. Failure of symptoms to resolve within three months has been found to be predictive of long-term persistence [74].

Many patients with MUS present intermittent symptoms and repeated episodes over one year [22] or occasional episodes warranting referrals over several years [75].

#### Severity of symptoms

High symptom severity and baseline disability, or the seriousness of the condition at baseline, seem to influence the prognosis. This has been found both in studies of pain and in patients with MUS [63, 65, 66].

Disability is already a central feature in the diagnostic criteria for disorders representing a spectrum of severity, e.g., psychiatric diagnoses such as depression and anxiety disorders [76]. As symptoms in themselves represent a spectrum of severity, the degree of disability may serve as a delimitation criterion to help distinguish between less severe and more severe conditions.

### Personal characteristics

#### Concurrent disorders

The prognostic value of common mental disorders is weak in patients presenting recent onset symptoms in primary care [63]. In patients with multiple symptoms categorised with either “recurrent or persistent symptoms” or “symptom disorders”, the presence of anxiety and/or depression is associated with future persistence of symptoms [62, 64]. This association also holds for co-existing physical disease [62].

#### Psychological factors

Psychological factors also play a role as risk indicators. Illness worry may be an important factor [61, 74, 77] along with more general aspects of negative illness perceptions [78], negative affect, causal attributions [72] and maladaptive coping, e.g., fear avoidance and catastrophizing [72, 79]. Finally, negative life events [72] and reported abuse during childhood predict poor outcome [62].

#### Demographic factors

Besides symptom and health characteristics, a few demographic factors have been investigated. Older age is a predictor of poor outcome in general [62, 64, 65]. Low education level and unmarried status (separated, widowed or divorced) indicate risk of symptom persistence in patients with high symptom scores [62].

### From prognostic information to classification

The factors described above do not contain natural or intuitive cut-points. Therefore, any assessment of prognosis will depend on a combination of several factors. We have selected three of the presented factors that merit particular emphasis: number of symptoms, number of bodily system and frequency of symptoms in the patient. These can be summarised as “**multiple symptoms, multiple systems and multiple times**”.

We propose to direct special attention to these three symptom factors for several reasons: (a) our experience with training GPs suggests that these factors are not routinely recognised in general practice despite their prominence in the research literature; (b) severity, concurrent disorders and demographic factors are prognostic factors for almost all conditions and not specifically to MUS; (c) assessment of psychological factors is a challenge in primary care because it requires special knowledge, skills and time, which may not be within easy reach in general practice. GPs tend to have a biased focus with a strong preference for biomedicine in their clinical assessment [80], and the recognition of these symptom characteristics is concordant with this mode of thinking. In Table 1, we provide qualitative descriptions of the way that patients may be classified using these three criteria.

**Table 1:** Proposed qualitative prognostic classification of symptoms based on *“multiple symptoms, multiple systems and multiple times”*

	Self-limiting symptoms	Recurrent or persistent symptoms ^a^	Symptom disorder
Number of symptoms	+ Few and related symptoms	++ Several symptoms (at same time or on different occasions)	+++ Many symptoms concurrently and/or over time
Number of body systems ^b^	+ One body system	++ One or more body systems	+++ Several body systems
Number of times	+ Few/infrequent	++ Several - intermittent or low-level persistence	+++ Many times or continuously
Risk of poor outcome	Low	Medium	High

^a^ Both “Recurrent or Persistent Symptoms” and “Symptom Disorder” meet the three criteria “multiple symptoms, multiple systems, multiple times”; they vary in the extent to which these criteria are met

^b^ For research, rigid criteria may apply; for clinical practice (as the aim is prognosis rather than formal diagnosis), “number of body systems” is applied more flexibly, e.g., grouping of symptoms by digestive, cardiovascular, genitourinary, etc., systems

Of the three categories in our prognostic classification, the second category “recurrent or persistent symptoms” is key for primary care. Although self-limiting symptoms are common, their good prognosis means that they can safely be managed within conventional consultations. Symptom disorder affects relatively few patients; most of these patients meet the criteria for psychiatric classification disorders, such as SSD, of at least moderate severity, and they may benefit from specialist or multidisciplinary treatment. Identifying patients with “recurrent or persistent symptoms” using criteria of multiple symptoms, multiple systems and multiple times has six potential advantages: (i) patients with increased likelihood of continuing or future symptoms are quickly identified, (ii) by emphasising patterns of symptoms rather than their nature, the category is easier to use in consultations where patients are struggling to have the severity of their symptoms heard [81], (iii) it implies a common problem with symptom processing [9] rather than direct causes for individual symptoms and so may shift the focus of diagnostic attention away from peripheral causes, (iv) the new approach does not exclude peripheral causes and can be used in parallel with further diagnostic assessment as indicated by new symptoms, (v) the new approach opens the door to “rational explanations” [44] and embraces both peripheral and central processes as causes of symptoms, and, finally, (vi) once more clearly defined, the criteria are sufficiently simple to use in both observational and interventional clinical research and yet may also serve as a diagnostic tool in daily clinical practice.

#### Next steps

We believe that the suggested classification could be used in routine care. Taking a prognostic approach, while remaining agnostic about aetiology, is likely to be acceptable for both doctors and patients. However, the implementation of a new classification approach will need investigations on its own in primary care populations.

The prognostic classification also highlights the need for primary care research to develop and evaluate appropriate management interventions for patients with recurring or persistent symptoms. Such interventions need to integrate both the biological dimension and the psychosocial dimension and are likely to include enhanced explanation and symptom management techniques while maintaining an eye on the horizon for hitherto unrecognised disease. The success, or otherwise, of a prognostic classification in primary care may depend on what happens elsewhere in health services. While open discussion with patients about prognostic categorisation may help limit testing and referral in healthcare systems where primary care has a strong gatekeeper function, it may need adoption across other specialties where patients are able to directly access multiple specialist opinions.

## Conclusion

Classification of symptoms into rigid categories of *organic* or *medically unexplained* is neither feasible nor helpful in primary care. Such classification may rather imply that symptoms are difficult to operationalize, investigate and manage. In this paper, we propose a new approach to classification of symptoms that places greater emphasis on prognostic factors. We argue that three specific criteria (multiple symptoms, multiple systems, multiple times) can act as simple classifiers into categories of *good*, *intermediate* and *poor* prognosis. This new classification may provide a rational framework for both clinical practice and future research.

## Abbreviations

BDS: Bodily distress syndrome
GP: General practitioner
IBS: Irritable bowel syndrome
MUS: Medically unexplained symptoms
SSD: Somatic symptom disorder
